# Expression of tumor necrosis factor α-induced protein 8 is upregulated in human gastric cancer and regulates cell proliferation, invasion and migration

**DOI:** 10.3892/mmr.2015.3690

**Published:** 2015-04-27

**Authors:** YANSEN LI, CHANGQING JING, YUEZHI CHEN, JINSHEN WANG, MINGLIANG ZHOU, XIN LIU, DONG SUN, LINJUN MU, LEPING LI, XIAOBO GUO

**Affiliations:** Department of Gastrointestinal Surgery, Shandong Provincial Hospital Affiliated to Shandong University, Jinan, Shandong 250021, P.R. China

**Keywords:** TNFAIP8, gastric cancer, proliferation, invasion, migration

## Abstract

Tumor necrosis factor α-induced protein 8 (TNFAIP8) has been associated with the tumorigenicity of various types of cancer, however, the expression of TNFAIP8 and its function in gastric cancer remain to be fully elucidated. Therefore, the present study examined the expression and biological function of TNFAIP8 in gastric cancer. The expression levels of TNFAIP8 were determined in 86 gastric cancer tissue samples and adjacent normal tissues using immunohistochemistry, and in four gastric cancer cell lines and GES-1 cells using reverse transcription-quantitative polymerase chain reaction. The expression of TNFAIP8 and its association with the tumor, node, metastasis (TNM) status and lymphatic metastasis of gastric cancer was evaluated. Furthermore, the functions of decreased expression levels of TNFAIP8 were analyzed in human gastric cancer cell lines. The expression of TNFAIP8 was significantly upregulated in the gastric cancer tissues and in the gastric cancer cell lines, and its expression levels were associated with the TNM staging and lymphatic metastasis. Furthermore, decreased expression of TNFAIP8 inhibited the growth, invasion and migration of gastric cancer cells. These data provided an innovative insight suggesting the downregulation of TNFAIP8 as a meaningful approach for treating human gastric cancer and other types of cancer. In addition, the expression levels of TNFAIP8 may be considered as a biomarker of gastric cancer progression.

## Introduction

Gastric cancer, although decreasing in incidence within the past few years, continues to be one of the serious threats to human health (1). Worldwide, gastric cancer is an aggressive tumor, leading to the fourth most common type of human malignancy and the second cause of death (2,3). Although surgical resection and adjuvant chemotherapy have progressed, and certain types of gastric cancer can be cured at an early stage (4), the majority of patients present at an advanced stage at diagnosis, and effective treatment methods are unavailable (5). Therefore, identifying an effective biomarker for early diagnosis and improving treatment strategies are required.

The novel candidate oncogene, tumor necrosis factor α-induced protein 8 (TNFAIP8), has received increasing attention. TNFAIP8, also termed NDED, GG2-1, SCCS2, SCC-S2 and MDC-3.13, is located in 5q23.1 and is involved in the malignancies of numerous types of tumor. Previously, the role of TNFAIP8 in the formation of a series of tumors has been determined (6–11). These results demonstrated that TNFAIP8 is involved in tumor progression and in the regulation of cell proliferation, invasion, migration, apoptosis and drug resistance among different types of tumor.

However, the expression of TNFAIP8 and its clinical significance in gastric cancer remain to be fully elucidated. In the present study, the expression of TNFAIP8 in gastric cancer was detected using immunofluorescence to confirm its cytoplasmic localization. Immunohistochemical, western blotting and reverse transcription-quantitative polymerase chain reaction (RT-qPCR) analyses were used to evaluate the expression of TNFAIP8 in gastric cancer tissues and cell lines, compared with adjacent normal tissues and GES-1 cells, and its relationship with clinicopathological characteristics and its prognostic roles were evaluated in 86 patients on long-term follow-up. Subsequent investigation involving knockdown of the TNFAIP8 gene was performedd to detect its potential regulatory mechanism and association with cell proliferation, invasion and migration.

## Patients and methods

### Patients and samples

Fresh samples of gastric cancer and adjacent normal tissues (four specimens) were collected for detection of the protein expression levels of TNFAIP8 via western blotting. A further 86 samples (46 males and 40 females; median age, 52; age range, 23–77) of gastric cancer tissues and adjacent normal tissues were obtained, between January 2007 and July 2008, from the Department of Pathology of Shandong Provincial Hospital (Shandong, China), which had undergone radical surgical therapy, according to the National Comprehensive Cancer Network Practice Guidelines (12). None of the samples had received preoperative treatment in the form of chemotherapy or radiotherapy. The tissues were collected from patients and healthy controls at the Provincial Hospital Affiliated to Shandong University, following the obtaining of informed consent from the patient’s family. The study was approved by the ethics committee of Shandong Provincial Hospital affiliated to Shandong University (Jinan, China). All patients had a confirmed diagnosis of gastric carcinoma by pathological examination following resection.

### Immunohistochemical (IHC) staining of tissues

For IHC staining, the tumor specimens were embedded in paraffin (Chemact (Liaoning) Petrochemicals Ltd., Liaoning, China) and 4-*µ*m thick sections were produced by the Department of Pathology of Shandong Provincial Hospital. Briefly, the sections were dewaxed according to the streptavidin-biotin-peroxidase complex (Zhong Shan Golden Bridge Biological Technology, Inc., Beijing, China) manufacturer’s instructions. Hydrogen peroxide (0.3%) and blocking serum (goat serum; Zhong Shan Golden Bridge Biological Technology, Inc.) were used (one blocking step; 30 min; 37°C) to inhibit endogenous non-specific substances. Following each blocking step, the tissues were washed with phosphate-buffered saline (PBS; Wuhan Boster Biological Technology, Ltd., Wuhan, China) for 5 min three times. The tissues were then incubated at 4°C overnight with TNFAIP8 rabbit anti-human polyclonal antibody (1:100; cat. no. 64988; Abcam, Cambridge, MA, USA). Following washing with PBS, the tissues were incubated with secondary goat anti-rabbit monoclonal antibody (1:1,000; cat. no. M080825; Zhong Shan Golden Bridge Biological Technology, Inc.) for 30 min at 37°C. Then tissues were stained with 3,3′-diaminobenzidine (Zhong Shan Golden Bridge Biological Technology, Inc.) and hematoxylin (Wuhan Boster Biological Technology, Ltd.) for 30 min at 37°C. The experiment was repeated three times.

### Immunofluorescence

Immunofluorescence was also performed to confirm the location of TNFAIP8 protein, using the following technique. The cell climbing piece was placed into a 24-well plate with 2,000 cells for 12 h. The cells were fixed with methanol for 5 min and then the climbing piece was washed with PBS for 5 min three times. The cells were then blocked using 3% bovine serum albumin (BSA; 300 *µ*l 10% BSA, 700 *µ*l PBS and 10 *µ*l Triton X-100) purchased from Wuhan Boster Biological Technology, Ltd. for 30 min. Cells were washed with PBS for 5 min three times, then were incubated at 37°C for 3 h with the TNFAIP8 rabbit anti-human polyclonal antibody (1:50). Subsequent to washing with PBS, the tissues were then incubated with the monkey anti-rabbit monoclonal secondary antibody (1:500; cat. no. A24221; Zhong Shan Golden Bridge Biological Technology, Inc.) for 1 h at 37°C. The liquid was then discarded, cells were stained with DAPI (Wuhan Boster Biological Technology, Ltd.). Subsequent to mounting, images were captured using an inverted fluorescence microscope (DP72; Olympus, Tokyo, Japan).

### Evaluation of IHC staining

The stained tissues were evaluated by two pathologists in a blinded-manner. Scores were assigned, dependent on the staining intensity and proportion, as previously described (13). Based on the intensity, the degrees were categorized as follows: 0, negative; 1, weak; 2, moderate; and 3, strong. The proportion of TNFAIP8 staining was scored using the following scale: 0, absent; 1, <25%; 2, 26–50%; 3, 51–75%; and 4, >75%. The final result was the sum of the intensity and proportion scores. IHC scores of <4 were considered to indicate low levels of expression, and those >4 were considered indicative of high levels of expression.

### Cell culture

The GES-1, MKN-28, SGC-7901 and MGC-803 cells were maintained in liquid nitrogen (Shanghai Jiayu Chemical Co., Ltd. Shanghai, China) in the Central laboratory of Shandong Provincial Hospital affiliated to Shandong University. The NCI-N87 human gastric cancer cell line was provided by the Institution of Digestive Surgery of Ruijin Hospital Affiliated to Shanghai Jiaotong University (Shanghai, China). The cells were cultured in RPMI-1640 medium (Sigma-Aldrich, St. Louis, MO, USA) supplemented with 10% heat-inactivated fetal bovine serum (FBS; GE Healthcare Life Sciences, Beijing, China), penicillin (100 U/ml) and streptomycin (100 mg/l) (Shanghai FMGBio Co., Ltd., Shanghai, China), in a humidified atmosphere containing 5% CO_2_ at 37°C.

### RT-qPCR

Total cellular RNA was isolated from the cells using TRIzol reagent (Invitrogen Life Technologies, Carlsbad, CA, USA), according to the manufacturer’s instructions. The RNA was reverse transcribed and the resulting cDNA samples were amplified by qPCR using a LightCycler 480 Real-Time PCR system (Roche Diagnostics, Shanghai, China) using gene-specific primers (Takara Bio, Inc., Dalian, China). In brief, 20 *µ*l PrimeScript™ RT reagent kit (DRR037A; Takara Bio, Inc., Shiga, Japan) containing 4 *µ*l 5X PrimeScript Buffer 2, 1 *µ*l PrimeScriptRT Enzyme Mix I, 1 *µ*l RT Primer Mix, 4 *µ*l RNase Free dH_2_O and 10 *µ*l RNA template. The RT-qPCR program comprised 37°C for 15 min followed by 85°C for 5 sec and 4°C for 30 min. A total of 2 *µ*l RT product (cDNA) was amplified by RT-qPCR using SYBR Green (DRR041A; Takara Bio, Inc.). The amplification conditions were as follows: 95°C for 30 sec, then 40 cycles at 95°C for 5 sec and 65°C for 30 sec. The sequences of the primer pairs were as follows: TNFAIP8, forward 5′-TTC CAT CAG GTG GAT TAT ACC TTTG-3′ and reverse 5′-AGG TGG CGC TGA ATG ATT TG-3′. The mRNA levels were normalized to that of GAPDH.

### Sequence design and vector construction

For small interfence (si)RNA treatment, siRNA to TNFAIP8 was synthesized by Shanghai Genechem Co., Ltd. The DNA target sequence for siRNA-TNFAIP8 (5′-CCA CCT TAA TAG ACG ACA CAA-3′) was designed, based on the core sequence of human TNFAIP8 cDNA. The cells, were transfected with the siRNA, according to the manufacturer’s instructions, and were observed under a microscope (DP72).

### Lentivirus transfection

For transfection, the SGC-7901 and MKN-28 gastric cancer cells were pre-cultured for 30 min at 37°C in 96-well plates at a density of 5×10^3^ cells per well. The cells were infected with the lentiviral vectors for 30 min at 37°C at different multiplicities of infection (10, 20, 50, 70 and 100; 50 selected as the optimal), when they were ~50–60% confluent. The transfection efficiency was observed using an inverted fluorescence microscope (DP72) after 72 h.

### Western blotting

The tissues and cells were washed in PBS, lysed (Beijing Solarbio Science & Technology Co., Ltd., Beijing, China) and harvested by centrifugation at 7,500 × g for 25 min. The protein concentration in the resulting lysate was evaluated using a Bicinchoninic Acid Protein Assay kit (Pierce Biotechnology, Inc., Rockford, IL, USA). Appropriate quantities of protein were then separated by electrophoresis on 12% Tris-glycine polyacrylamide gels (Zhong Shan Golden Bridge Biological Technology, Inc.) and transferred onto nitrocellulose membranes (Zhong Shan Golden Bridge Biological Technology, Inc.). The membranes were blocked with 10% skimmed milk powder and then incubated overnight at 4°C with the rabbit anti-human TNFAIP8 primary antibody (1:100; cat. no. 64988, Abcam). On the following day, the membranes were washed three times with Tris-buffered saline with 1% Tween 20 (Wuhan Boster Biological Technology, Ltd.) for 10 min and incubated with the corresponding goat anti-rabbit monoclonal secondary antibody (1:2,000) at 37°C for 1 h. Following washing three times for 10 min, the bound secondary antibody was detected using an enhanced chemiluminescence system (Pierce Biotechnology Inc.). The protein levels were normalized against β-actin.

### Colony formation assay

The gastric cancer cells were digested into single suspension cells and 1×10^3^ cells were plated in 60 mm plates with 4 ml complete culture medium (5% FBS and 1%penicillin/streptomycin). The plates were cultured at 37°C in 5% CO_2_ for 10 days. Colonies comprising at least 50 cells were considered to be statistically significant. The results are presented as the mean ± standard deviation from five randomly selected fields.

### Cell counting kit (CCK)-8 assay

The gastric cancer cells (8×10^2^ cells) were incubated in 96-well plates with 100*µ*l 10% FBS, and were continuously incubated for 24, 48, 72, 96 and 120 h at 37°C in 5% CO_2_. The number of cells were estimated using a CCK-8 (cat. no. C0038; Dojindo Molecular Technologies, Kumamoto, Japan). Briefly, 10 *µ*l CCK-8 was added to each well and, following incubation for 1 h, the absorbance at 450 nm was measured to calculate the number of cells. The analysis of each cell type were repeated six times independently.

### Invasion and migration assay

For invasion assays, 3×10^5^ cells were plated into 200 *µ*l RPMI-1640 medium in the upper chamber of a Transwell, which was separated from the lower chamber by a 50 *µ*l Matrigel-coated membrane (24-well insert; 8-*µ*m pore size; Corning Costar, Corning, NY, USA). For the migration assay, the membranes were not coated with Matrigel, although the culture conditions were the same as in the invasion assay (37°C, 5% CO_2_, 24 h for invasion assay, 36 h for migration). Finally, for the two assays, the membranes were removed and stained with hematoxylin, and images were captured using an SMZ171 microscope (Olympus).

### Statistical analysis

Statistical analysis was determined using SPSS 18.0 software (SPSS, Inc., Chicago, IL, USA). The protein and mRNA expression levels of TNFAIP8 in human gastric cancer cell lines and tissues were expressed as the mean ± standard deviation of at least three independent experiments. The χ^2^ and Fisher’s exact tests were used for the analysis of categorical variables. P<0.05 was considered to indicate a statistically significant difference.

## Results

### TNFAIP8 is upregulated in gastric cancer tissues and cell lines

**Figure 1:**
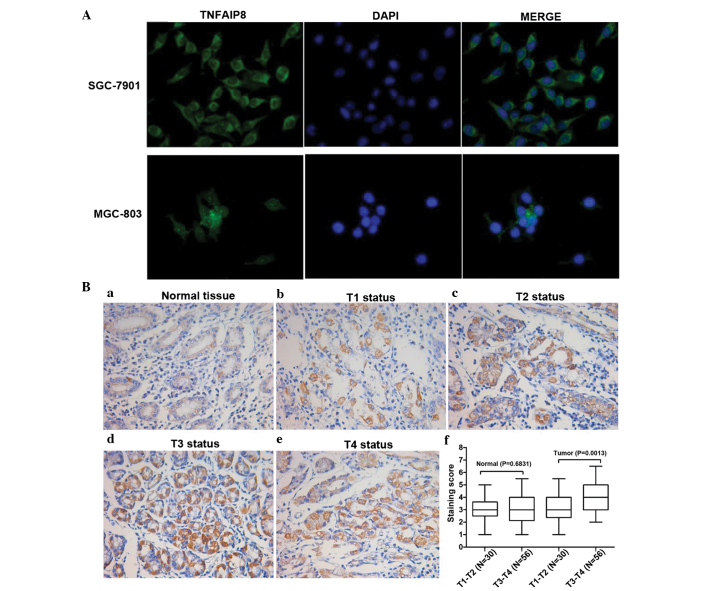
Expression of TNFAIP8 is upregulated in gastric cancer tissues samples. (A) Expression of TNFAIP8 in SGC-7901 and MGC-803 gastric cancer cells (magnification, ×400). Weaker immunohistochemical staining was observed in the (Ba) normal tissues compared with the (Bb-Be) primary carcinoma tissues at different stages of invasion (magnification, ×400). (Bf) Comparison of staining scores between the T1+T2 and T3+T4 tissues. Data are expressed as the mean ± standard deviation. TNFAIP8, tumor necrosis factor α-induced protein 8 DAPI, 3,3′-diaminobenzidine.

Although TNFAIP8 has been detected, whether its expression is associated with tumor invasion remains to be fully elucidated (14). Correlations between the expression of TNFAIP8 and various clinicopathological features are listed in Table I. In the present study, the protein levels of TNFAIP8 were examined using IHC staining in normal tissues and tumor tissues, varying in depth of invasion. The tissues from 56 patients with gastric cancer of T3+T4 status were compared with 30 patients with gastric cancer patients of T1+T2 status. Within all the tissues, the intensity of TNFAIP8 staining was observed in the following order: T3+T4>T1+T2 (Fig. 1Ba–Bf). The expression levels of TNFAIP8 were significantly associated with tumor status (P<0.05; Table I; Fig. 1B; n=86). Furthermore, the expression of TNFAIP8 in the tumor tissues was higher than in the adjacent normal tissues. In addition, the staining points of the normal tissue indicated no significant difference in the levels of TNFAIP8 between the T3+T4 and T1+T2 groups, however TNFAIP8 was upregulated in the tumor tissues from T3+T4 patients, compared with those from T1+T2 patients (Fig. 1Bf). Despite the requirement for further investigations to confirm and develop this IHC data, these results revealed that TNFAIP8 was commonly enhanced in gastric cancer tissues, compared with normal tissues or deeper invasion tissues. This suggested that TNFAIP8 can be used as a sole prognostic value for metastasis and is expected to become a potential target for the treatment of gastric cancer.

**Figure 2:**
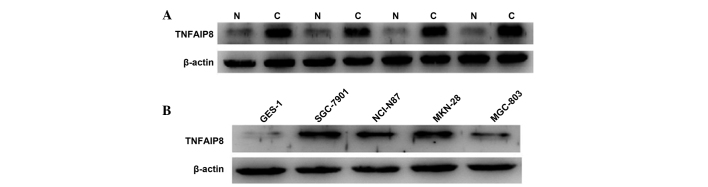
Western blot analysis of TNFAIP8 in gastric cancer tissues and cell lines. (A) Differences in the expression of TNFAIP8 between the cancer tissues (C) and adjacent normal tissues (N). (B) Expression levels of TNFAIP8 in different gastric cancer cell lines were compared with normal gastric mucosal cells. (P<0.05). TNFAIP8, tumor necrosis factor α-induced protein 8.

The overexpression of TNFAIP8 protein in gastric carcinoma tissues compared with normal tissues was demonstrated, which was in accordance with the results of a previous study (14). Furthermore, the four gastric cancer cell lines exhibited higher expression levels of TNFAIP8 than the GES-1 cells, and the SGC-7901 and MKN-28 cells exhibited relatively high levels of expression in the four cancer cell lines (Fig. 2).

### TNFAIP8 knockdown inhibited gastric cancer cell proliferation, invasion and migration

**Figure 3:**
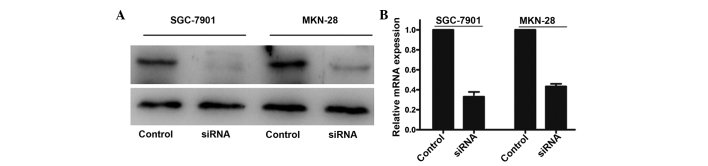
Efficiency of TNFAIP8 knockdown in SGC-7901 and MKN-28 cell lines. (A) Western blot analysis indicated that the protein expression of TNFAIP8 following treatment with siRNA was significantly suppressed compared with the negative control. (B) Reverse transcription-quantitative polymerase chain reaction revealed that treatment with siRNA markedly decreased the mRNA levels of TNFAIP8 in the gastric cancer cells. siRNA, small interference RNA.

In order to examine the biological role of TNFAIP8 in gastric cancer cells, the present study knocked down the TNFAIP8 gene in SGC-7901 and MKN-28 cell lines, which exhibited higher protein expression levels of TNFAIP8, and performed qRT-PCR and western blot analyses to confirm the knockdown efficiency. The results demonstrated that treatment with siRNA markedly decreased the protein and mRNA expression levels of TNFAIP8 (Fig. 3).

**Figure 4:**
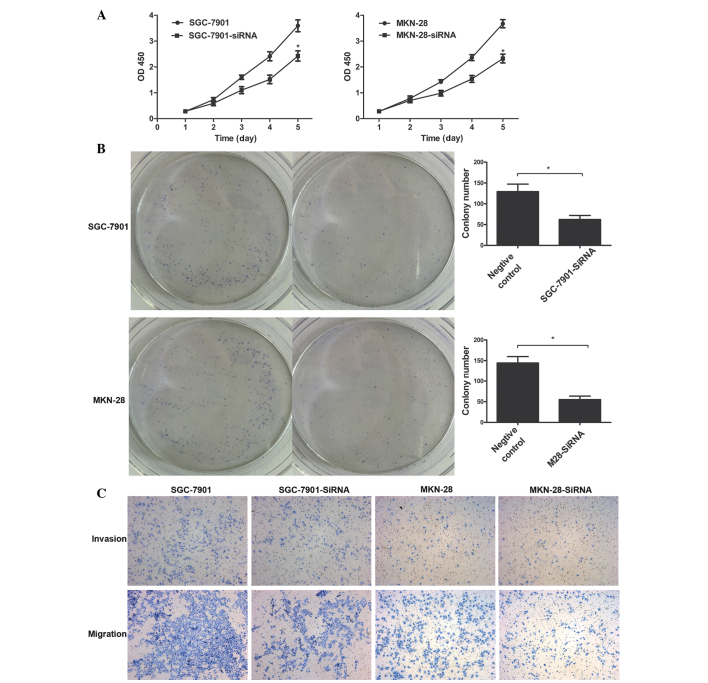
TNFAIP8 knockdown inhibits proliferation, colony formation, invasion and migration in gastric cancer cell lines. Data are expressed as the mean ± standard deviation. (A) Counting kit-8 assay results demonstrated that decreased expression levels of TNFAIP8 inhibited proliferation in the SGC-7901 and MKN-28 cell lines. (B) Colony formation assay results revealed that the number of cells decreased significantly in the TNFAIP8 siRNA group compared with the negative control group. (C) Transwell assay demonstrated that cells in the TNFAIP8 siRNA group had reduced invasive and migratory capacities compared with the negative control group. ^*^P<0.05. TNFAIP8, tumor necrosis factor α-induced protein 8; OD, optical density; siRNA, small interference RNA.

Tumorigenicity was significantly suppressed in the TNFAIP8-transfected cells. The role of TNFAIP8 in tumor aggression was apparent in the negative control groups, compared with the TNFAIP8-siRNA treatment group (Fig. 4A and B; P<0.01). In addition, decreased expression levels of TNFAIP8 in the SGC-7901 and MKN-28 cells significantly inhibited the invasion and migration of these cells, compared with the invalid siRNA-treated cells (P<0.05; Fig. 4C). Taken together, these data suggested that TNFAIP8 was involved in promoting the progression of gastric cancer.

## Discussion

Gastric cancer, a serious threat to human health, is a complex disease affected by numerous factors. In the majority of cases, the point at which gastric cancer is diagnosed is at a stage, which is incurable, and the overall prognosis is poor (15). Numerous studies have investigated the pathogenesis and prognosis of gastric cancer (16–18). The examination of levels of epidermal growth factor, cyclin E, p27, E-cadherin, CD44v6, matrix metalloproteinase (MMP-1 and tissue inhibitor of metalloproteinase-1, human epidermal growth factor receptor-2 and vascular endothelial growth factor (VEGF) (19) may be of important significance for determining the prognosis and individualized treatment of patients with gastric cancer. However, the mechanism of gastric cancer remains to be fully elucidated, and no single index can successfully predict or prolong the survival rate of patients with gastric cancer. Therefore, further investigations of the mechanism of gastric carcinogenesis, effective predictors and treatment are required.

The expression of TNFAIP8 has been identified in various types of human cancer (6–11). However, prior to the present study, the expression of TNFAIP8 and its functions associated with gastric cancer remained to be elucidated. To investigate this, the present study firstly corroborated the protein levels of TNFAIP8 in gastric cancer tissues compared with corresponding adjacent normal tissues using immunohistochemistry, western blotting and qRT-PCR. The results demonstrated that the protein levels of TNFAIP8 in the gastric cancer tissues were significantly higher than those of the matched adjacent normal tissues, and there was a close correlation between TNFAIP8 positivity, tumor, necrosis, metastasis stage, and lymph node metastasis. These results were in agreement with previous studies and indicated that TNFAIP8 may be involved in the progression of gastric cancer (14,19). The immunohistochemcal staining in the gastric cancer tissues and corresponding normal tissues were also compared, which indicated higher staining scores in the T3–T4 cancer group compared with the T1–T2 cancer group. These data indicated that TNFAIP8 is of predictive value for invasion and metastasis in gastric cancer and provide a novel insight for the estimation of the progression of gastric cancer. Collectively, these findings indicated that TNFAIP8 was associated with gastric cancer and can be considered as an oncogene.

However, it is difficult to determine whether downregulation of TNFAIP8 also affects gastric cancer cell tumorigenesis. To further examine the expression of TNFAIP8 in gastric cancer and the correlation of TNFAIP8 with the biological behaviors of gastric cancer cells, the present study analyzed the expression levels of TNFAIP8 in normal gastric mucosa epithelial cells and in four differential gastric cancer cell lines using western blotting. The expression levels of TNFAIP8 in the gastric carcinoma cells were significantly higher compared with those in the GES-1 cells. In order to investigate the functions of TNFAIP8 in tumor formation, the present study inhibited its function via siRNA treatment in the MKN-28 and SGC-7901 cell lines, which exhibit higher expression levels of TNFAIP8. The results revealed that knockdown of TNFAIP8 caused a significant inhibition of cell proliferation and colony formation ability, which were consistent with the immunohistochemical data and with the results of previous studies (8,14,20).

Cell migration promotes several biological processes (21) and enhanced activation of cell migration results in tumor metastasis, which is the predominant factor affecting survival rates (22). In the present study, the cell invasion and migration assay demonstrated that TNFAIP8 knockdown inhibited cell invasion and migration in the two types of gastric cancer cells. These data provided evidence to indicate that TNFAIP8 is important in cell invasion and metastasis. In support of this findings, Zhang *et al* (11) revealed that downregulation in the expression of TNFAIP8 decreased lung metastasis by inhibiting MMP1 and MMP2 metastasis-associated molecules in tumor cells, and VEGFR receptor 2 in endothelial cells.

In conclusion, the results of the present study indicated that the protein and mRNA expression levels of TNFAIP8 in gastric carcinoma tissues and cells were significantly higher compared with those in normal tissues and cells. Furthermore, TNFAIP8 was identified as a novel independent risk factor for predicting the prognosis of patients. Accordingly, TNFAIP8 IHC scores may be used as a novel diagnostic biomarker for determining the risk of metastasis and prognosis. The results of the present study demonstrated that TNFAIP8 acted as a functional oncogene protein in gastric tumorigenesis and was involved in promoting lymph node metastasis. The results provide further evidence that, in addition to enhancing tumorigenesis in other types of tumor, TNFAIP8 may be involved in promoting cell proliferation, invasion and migration. However, the TNFAIP8-mediated mechanisms of cell proliferation, invasion and migration remain to be elucidated. As TNFAIP8 is induced by the activation of the nuclear factor (NF)-kB transcription factor, TNFAIP8 may regulate cell function via the NF-kB signal transduction pathway. Additional investigations are currently being performed to attempt to establish TNFAIP8 as a potential therapeutic target.

## Figures and Tables

**Table I:** Comparison between the expression of TNFAIP8 in gastric cancer tissues and clinicopathological characteristics.

Characteristic	Number of patients (n)	TNFAIP8 expression level		P-value
		High, n (%)	Low, n (%)	
Tumor status				0.033
T1–T2	30	12 (40.00)	18 (60.00)	
T3–T4	56	34 (60.71)	22 (39.29)	
Lymph node metastasis				0.030
Negative	62	33 (53.22)	29 (46.77)	
Positive	24	19 (79.17)	5 (20.83)	

TNFAIP8, tumor necrosis factor α-induced protein 8.
